# Nebivolol protects the liver against lipopolysaccharide-induced oxidative stress, inflammation, and endoplasmic reticulum–related apoptosis through *Chop* and *Bip*/*GRP78* signaling

**DOI:** 10.1007/s00210-024-02990-3

**Published:** 2024-02-14

**Authors:** Onur Unal, Yalcin Erzurumlu, Halil Asci, Berivan Gunduru Acar, Mehmet Bedir, Ozlem Ozmen

**Affiliations:** 1 Department of Infectious Diseases and Clinical Microbiology, Faculty of Medicine, Suleyman Demirel University, Isparta, Turkey; 2 Department of Biochemistry, Faculty of Pharmacy, Suleyman Demirel University, Isparta, Turkey; 3 Department of Pharmacology, Faculty of Medicine, Suleyman Demirel University, Isparta, Turkey; 4 Department of Biochemistry, Faculty of Medicine, Suleyman Demirel University, Isparta, Turkey; 5 Department of Pathology, Faculty of Veterinary Medicine, Burdur Mehmet Akif Ersoy University, Burdur, Turkey

**Keywords:** Nebivolol, Liver, Endoplasmic reticulum stress, Inflammation, *Chop*, *Bip*

## Abstract

This study aimed to examine the protective role of nebivolol (NEB) on liver tissue against the lipopolysaccharide (LPS)-induced sepsis model in rats by targeting endoplasmic reticulum (ER) stress–related binding immunoglobulin protein (*Bip*), CCAAT-enhancer-binding protein homologous protein (*Chop*) signaling pathways. Four groups, each comprising eight rats, were established: control, LPS, LPS + NEB, and NEB. Biochemical analyses included total oxidant status (TOS), serum aspartate transaminase (AST), and alanine aminotransferase (ALT) levels. Additionally, genetic assessments involved *Chop* and *Bip*/*GRP78* mRNA expression levels, while histopathological examinations were conducted. Immunohistochemistry was used to determine interleukin-1 beta (IL-1 β) and caspase-3 levels. The LPS group exhibited significantly higher AST, ALT, oxidative stress index, and TOS levels compared to the control group. Moreover, the LPS group demonstrated markedly increased *Chop* and *Bip*/*GRP78* mRNA expression compared to the control group. Immunohistochemical analysis of the LPS group revealed significant upregulation in IL-1β and caspase-3 expressions compared to the control group. Additionally, the LPS group showed significant hyperemia, mild hemorrhage, and inflammatory cell infiltrations. Comparatively, the LPS+NEB group exhibited a reversal of these alterations when compared to the LPS group. Collectively, our findings, suggest that NEB holds promise as a treatment in conditions where oxidative damage, inflammation, and ER stress–related apoptosis play significant roles in the pathogenesis.

**Graphical abstract:**

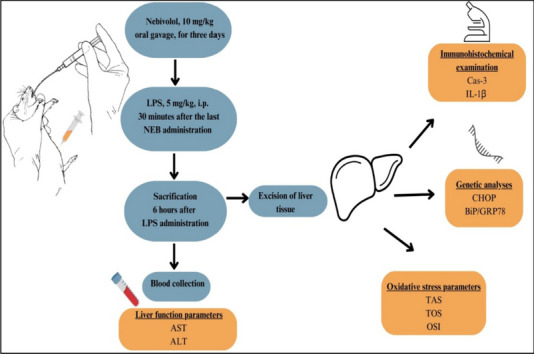

**Supplementary Information:**

The online version contains supplementary material available at 10.1007/s00210-024-02990-3.

## Introduction

Sepsis, an inflammatory response throughout the body triggered by infection, can progress to septic shock and multi-organ dysfunction, leading to severe complications with high morbidity rates. These complications encompass metabolic disorders, including impaired glucose homeostasis, increased energy consumption, and a negative nitrogen balance (Iwashyna et al. 2012). The liver, owing to its pivotal functions in synthesis, detoxification, energy production, and storage, plays a crucial role in combatting sepsis, a condition characterized by severe inflammation (Woźnica et al. 2018).

The liver serves a crucial role in eliminating lipopolysaccharide (LPS) from systemic circulation. LPS, a component found in the outer membrane of gram-negative bacteria, contributes to inflammation triggered by bacterial infections (Deng et al. 2013). LPS exposure can induce lipid peroxidation and oxidative damage in specific DNA segments and other proteins, primarily through the generation of reactive oxygen species (ROS), like superoxide, hydroxyl radicals, and hydrogen peroxide (Brenner et al. 2013). Furthermore, an elevation in metabolic factors, such as cytokines and hormones, during various pathological processes has been shown to provoke oxidative stress, further disrupting metabolic functions and intensifying inflammation. Notably, levels of ROS and intracellular calcium (Ca^2+^) signals play critical roles as messengers in the intricate relationship between oxidative stress and inflammation (Zhang 2010).

The endoplasmic reticulum (ER) and mitochondria engage in close interaction within a dynamic network, generating ROS and Ca^2+^ signals (Zhang and Kaufman 2008). The ER plays a pivotal role in various metabolic activities such as protein synthesis and transport, protein quality control, glycosylation, and intracellular Ca^2+^ homeostasis (Urra et al. 2013). External stressors affecting ER Ca^2+^ homeostasis can initiate “ER stress” leading to the accumulation of improperly folded proteins within the cell (Liu et al. 2011). Additionally, inflammatory cytokines like interleukin-1 beta (IL-1β) and tumor necrosis factor-alpha (TNF-α) can induce an acute-phase response in the liver, triggering ER stress in hepatocytes (Zhang 2010). These cytokines generate Ca^2+^ signals in the ER and heighten ROS accumulation through multiple mitochondrial metabolic pathways (Zhang 2010). Increased cytosolic calcium levels can accelerate mitochondrial metabolism, elevating ROS production. Mitochondrial ROS, in turn, can prompt increased calcium release from the ER, leading to harmful ROS accumulation (Zhang 2010). To counteract ROS-induced damage, the antioxidant defense system works to mitigate cellular damage. ER stress is closely associated with oxidative stress and the inflammatory response. Dysfunctions in any of these pathways can affect others, thus influencing cellular adaptations (Zhang and Kaufman 2008; Zhang 2010). Furthermore, studies have demonstrated that LPS, a potent inducer of bacterial infection–triggered inflammation, induces oxidative stress in liver tissue and instigates apoptotic processes related to ER stress (Zhang and Kaufman 2008; Zhang 2010).

Understanding the intricate connection between ER stress and mitochondrial dynamics underscores their pivotal roles in cellular homeostasis and determining cell fate. ER stress significantly influences mitochondrial function, and conversely, both organelles interconnect through various signaling pathways. Different stimuli can trigger ER stress known to culminate in apoptotic cell death across multiple cell types due to its prolonged duration (Liu et al. 2011). Moreover, ER stress escalation elevates levels of binding immunoglobulin protein (*Bip*), the primary ER chaperone protein. Among the key pro-apoptotic transcription factors in ER stress is the *Chop* protein, a member of the DNA binding transcription factor family (Liu et al. 2011; Malhi and Kaufman 2011; Kopp et al. 2019). Activated *Chop* phosphorylates BH3 proteins situated on the mitochondrial membrane, part of the Bcl2 protein family, thereby suppressing the anti-apoptotic activities of *Bcl2* or *Bcl-XL*. Furthermore, *Chop* plays a dual role in ER stress by inhibiting apoptosis directly, leading to an increased *Bcl-2*/*Bax* ratio (Nasiri-Ansari et al. 2021).

Nebivolol (NEB) stands as a long-acting, third-generation β1 adrenergic blocker, exhibiting distinct pharmacodynamic properties from other beta-blockers in its class. NEB showcases vasodilatory and antioxidative effects observed in animal models and human studies (Kamp et al. 2010; Refaie et al. 2018). Studies suggest that NEB’s antioxidant effects are linked to reduced ROS levels produced by the nicotinamide adenine dinucleotide phosphate (NADPH) oxidase enzyme system. The significant antioxidant properties of NEB and its metabolites have garnered interest for potential clinical applications in disorders affecting various systems, especially the cardiovascular system (Munzel and Gori 2009).

This study aims to evaluate the effects of NEB on various pathways in liver tissue, including ER stress, oxidative stress, inflammation, and apoptotic processes, utilizing a rat model of LPS-induced sepsis.

## Materials and methods

### Animals and ethical approval

All the experiments were conducted following the guidelines for animal research as per the Animal Research: Reporting in Live Experiments (ARRIVE 2.0) guidelines and received approval from the Local Committee on Animal Research of Suleyman Demirel University (approval number: 11.09.2020-06/18).

A total of 32 Wistar albino rats, weighing between 250 and 350 g, were utilized in this study. Before commencing the experiment, a veterinarian conducted health assessments on the rats to ensure their well-being. They were provided with a standard commercial diet (Korkuteli Yem, Antalya, Turkey) and housed in an environment maintaining a 12:12-hour light/dark cycle, with a constant temperature ranging between 21 and 22 °C and a humidity level between 60 and 65%. No death was observed throughout the experimental period.

### Experimental design

Four groups were randomly assigned: control (*n* = 8), LPS (*n* = 8), LPS + NEB (*n* = 8), and NEB (*n* = 8).

- *Control group*: The rats received 1 ml of saline by oral gavage for 3 days. Thirty minutes after the last oral saline administration, 1 ml of saline was injected intraperitoneally (i.p.) into the right inguinal area.
- *LPS group*: After 3 days of administering 1 ml of saline by oral gavage, rats were injected i.p. with 5 mg/kg LPS (048K4126, Sigma Aldrich, USA) dissolved in saline into the right inguinal area, 30 min after the last oral saline (Samuvel et al. 2016).
- *LPS + NEB group*: The rats received NEB (Nexivol, Abdi İbrahim, Isparta, Turkey) by oral gavage at a dose of 10 mg/kg for 3 days, diluted with saline. Thirty minutes after the final oral NEB administration, 5 mg/kg LPS was injected i.p. into the right inguinal region (Dursun et al. 2018).
- *NEB group*: The rats were orally administered 10 mg/kg NEB for 3 days. Thirty minutes following the last oral NEB administration, 1 ml of saline was injected i.p.

Six hours following LPS injection, all rats were anesthetized with 80-100 mg/kg ketamine (Ketasol, Richter Pharma AG, Austria) and 8-10 mg/kg xylazine solution (Xylazinebio, Bioveta, Czech Republic). Subsequently, blood samples were taken from the rats’ inferior vena cava via abdomen incisions following the administration of anesthesia. Half of the excised liver tissues were wrapped in aluminum foil and frozen at − 20 °C for further analysis. The remaining hepatic tissues were harvested for histological and immunohistochemical and fixed in 10% neutral formalin solution.

### Biochemical testing

#### Analysis of blood parameters

The rat blood was collected into gel-filled tubes and centrifuged at 3000 rpm for 10 min to obtain serum. After centrifugation, the serum was divided into three parts and stored at − 80 °C until analysis. Serum aspartate transaminase (AST) and alanine aminotransferase (ALT) levels were analyzed spectrophotometrically using a suitable kit and a Beckman Coulter AU 5800 autoanalyzer (Beckman Coulter, USA).

#### Parameters for measuring oxidative stress

The Ultra Turrax Janke&Kunkel T-25 homogenizer (IKA®-Werke, Germany) was used to homogenize hepatic tissue samples for oxidant-antioxidant analyses. Commercial kits (Rel Assay Diagnostics, Gaziantep, Turkey) and spectrophotometry (Beckman Coulter, USA) were used to assess total antioxidant status (TAS) and total oxidant status (TOS). The formula OSI = TOS/TAS was used to compute the oxidative stress index (OSI) (Altindag et al. 2008). The antioxidant effect of the sample against potent free radical reactions of hydroxyl radicals was calculated by measuring the change in absorbance at 660 nm. The data are reported in millimolar equivalents of Trolox per liter (Erel 2004). Using hydrogen peroxide, the test was calibrated, and the finding was represented in micromolar equivalents of hydrogen peroxide per liter (μmol H_2_O_2_ Eqv/L) (Erel 2005).

### RNA isolation and reverse transcription-quantitative polymerase chain reaction (RT-qPCR) analysis protocol

RNA was extracted with the AurumTM Total RNA Mini Kit Reagent Monarch Total RNA isolation kit (Bio-Rad Laboratories, Hercules, CA). Using a MySPEC microvolume spectrophotometer (VWR), RNA concentration and purity were measured. Using the iScript cDNA Synthesis Kit (Bio-Rad Laboratories, Hercules, CA), a microgram of RNA was translated into cDNA according to the manufacturer’s instructions. iTaq Universal SYBR Green Supermix was used to conduct RT-PCR amplification (Bio-Rad Laboratories, Hercules, CA). Primers to be used for amplification were designed through the NCBI website (Table 1). For each PCR, triplicate cDNA samples were analyzed. The GAPDH gene was utilized to standardize the data. The following PCR conditions were used: 5 min of denaturation at 95 °C, followed by 40 cycles of 12 s at 95 °C and 35 s at 60 °C. Total reaction volume was 25 µl, and 100 ng of cDNA served as the template. The comparative 2^−ΔΔCt^ method was employed to quantify relative gene expression. The melting curve was used to test the specificity of the PCR product amplification, and the findings were shown as a fold change.

**Table 1:** Primary sequences, product size and accession numbers of genes

Genes	Primary sequence	Product size	Accession number
GAPDH (HouseKeeping)	F: CAAGGTCATCCCAGAGCTGAA	340 bp	NM_017008.4
	R: CATGTAGGCCATGAGGTCCAC		
CHOP	F: TGGAAGCCTGGTATGAGGATCTG	175 bp	XM_006241445.4
	R: GAGGTGCTTGTGACCTCTGCTG		
BIP	F: TGTGACTGTACCAGCTTACTTC	149 bp	NM_013083.2
	R: TCTTCTCTCCCTCTCTCTTATCC		

*F* forward, *R* reverse, *GAPDH* glyceraldehyde-3-phosphate dehydrogenase, *CHOP* CCAAT-enhancer-binding protein homologous protein, *BIP* binding immunoglobulin protein

### Histopathological examination

The collected liver samples were fixed in a 10% neutral formalin solution. After fixation, tissue samples were routinely processed using fully automated tissue processing equipment (Leica ASP300S, Wetzlar, Germany) and embedded in the paraffin wax. After cooling, 5-µm-thick sections were cut from paraffin blocks with a rotary microtome (Leica RM2155, Leica Microsystems, Wetzlar, Germany). The prepared slides were examined under a light microscope after being stained with hematoxylin-eosin (HE). Histopathological evaluation was conducted on a total of 4 liver sections from each rat.

A semi-quantitative grading system was used to identify the progression of the injury based on a scale of 0–3, whereby 0 indicates no discernible injury, 1 slight immune cell infiltration, 2 hepatocyte ballooning/degeneration, and 3 loss of hepatic structure, increased apoptotic bodies/massive immune cell infiltration (Chen et al. 2012).

#### Immunohistochemical assessment

Two series of sections were taken from each block and subjected to immunohistochemical staining for caspase-3 (Cas-3 (E-8):sc-7272) and IL-1β (IL-1 β (11E5):sc-52012) (Santa Cruz, Texas, USA) expression using the streptavidin-biotin technique. Immunohistochemistry involved the application of a biotinylated secondary antibody and a streptavidin-alkaline phosphatase conjugate on sections treated with primary antibodies for 60 min. The EXPOSE Mouse and Rabbit Specific HRP/DAB Detection IHC Kit (ab80436) (Abcam, Cambridge, UK) served as the secondary antibody, with diaminobenzidine (DAB) as the chromogen. Negative controls utilized an antigen dilution solution instead of primary antibodies.

A semi-quantitative analysis utilizing a grading scale from 0 to 3 assessed cell immunohistochemistry reactivity with markers: 0, negative; 1, focal weak; 2, diffuse weak; 3, diffuse strong. For immunohistochemical examination, slides were analyzed independently for each antibody. In each four sections, 10 distinct areas were evaluated using ImageJ (National Institutes of Health, Bethesda MD). The Database Manual Cell Sens Life Science Imaging Software System performed microphotography (Olympus Co., Tokyo, Japan). The grading of the slides for both histopathological and immunohistochemical examinations was performed by a researcher who was unaware of the sample group identities from another university.

### Statistical analyses

Biochemical, genetic, histopathological, and immunohistochemical data were analyzed using SPSS 20.0 (SPSS Inc., Chicago, IL, USA). The data were examined across groups using post hoc One-way ANOVA and LSD testing. The mean ± standard deviations (SD) of the variables were reported. The significance threshold was deemed to be *p* < 0.05.

## Results

### Biochemical results

#### NEB reverses the increase in AST and ALT levels due to LPS-induced liver damage

When compared to the control group, the LPS group displayed a significant increase in AST and ALT levels (*p* < 0.001). Moreover, the LPS+NEB group exhibited significantly lower AST and ALT levels in comparison to the LPS group (*p* < 0.001). Additionally, the NEB group showed a significant decrease in all parameters compared to the LPS group (*p* < 0.001) (Table 2).

**Table 2:** Biochemical changes in the blood of the experiment groups

Groups	AST		ALT	
	Mean ± SD	*p* value	Mean ± SD	*p* value
Control	105.50 ± 23.07		48.12 ± 7.06	
LPS	626.88 ± 261.93***	< 0.001***	443.63 ± 230.76^a^	< 0.001***
LPS+NEB	190.81 ± 87.34^###^	< 0.001^###^	88.72 ± 38.38^b^	< 0.001^###^
NEB	70.04 ± 9.67^###^	< 0.001^###^	39.86 ± 4.90^b^	< 0.001^###^

Values are presented as means ± SD. The relationships between groups and results of biochemical markers are assessed by one-way ANOVA test (post hoc LSD test)

*AST* aspartate aminotransferase, *ALT* alanine aminotransferase, *LPS* lipopolysaccharide, *NEB* nebivolol

^*^Compared to control group. * *p* ≤ 0.05, **p ≤ 0.01, ***p ≤ 0.001

^#^Compared to the LPS group, ^#^*p* ≤ 0.05, ^##^*p* ≤ 0.01, ^###^*p* ≤ 0.001

#### NEB reduces oxidative stress induced by LPS in liver tissue

TAS levels were significantly lower in the LPS group compared to the control group (*p* = 0.007). Additionally, although TAS levels appeared higher in the LPS+NEB group compared to the LPS group, this difference did not reach statistical significance. Moreover, TAS levels showed a significant increase in the NEB group compared to the LPS group (*p* = 0.004).

The LPS group demonstrated significantly higher OSI levels compared to the control group (*p* = 0.002). Both the LPS+NEB (*p* = 0.018 for TOS and *p* = 0.001 for OSI) and NEB groups (*p* = 0.031 for TOS and *p* < 0.001 for OSI) displayed notably lower TOS and OSI levels than the LPS group (Table 3).

**Table 3:** TAS, TOS, and OSI values in liver tissue of groups

Groups	TAS (mmol trolox equivalents/L)		TOS (µmol H_2_O_2_ equivalents/L)		OSI index	
	Mean ± SD	*p* value	Mean ± SD	*p* value	Mean ± SD	*p* value
Control	1.71 ± 0.12		20.08 ± 1.81		1.18 ± 0.15	
LPS	1.49 ± 0.24	0.007**	22.11 ± 3.34		1.50 ± 0.27	0.002**
LPS+NEB	1.64 ± 0.08		18.64 ± 2.41	0.018^#^	1.13 ± 0.13	0.001**
NEB	1.73 ± 0.07	0.004^##^	18.99 ± 3.14	0.031^#^	1.09 ± 0.18	<0.001***

Values are presented as means ± SD. The relationships between groups and results of oxidative stress markers are assessed by one-way ANOVA test (post hoc LSD test)

*TAS* total antioxidant status, *TOS* total oxidant status, *OSI* oxidative stress index, *LPS* lipopolysaccharide, *NEB* nebivolol

^*^Compared to control group. *p ≤ 0.05, **p ≤ 0.01, ***p ≤ 0.001

^#^Compared to the LPS group, ^#^*p* ≤ 0.05, ^##^*p* ≤ 0.01, ^###^*p* ≤ 0.001

### RT-qPCR relative messenger RNA (mRNA) expression analysis

#### NEB attenuates LPS-induced ER stress

**Fig. 1:**
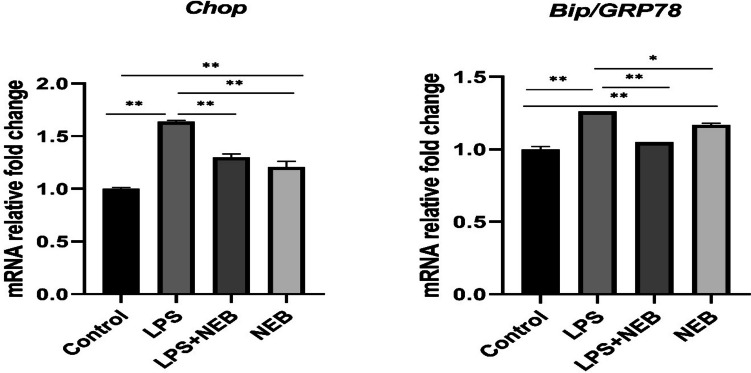
Relative mRNA expression levels of *Chop* and *Bip*/*GRP78*. Values are expressed as the mean ± SD. *Chop*: CCAAT-enhancer-binding protein homologous protein, *Bip*: binding immunoglobulin protein, LPS: lipopolysaccharide, NEB: nebivolol. *p < 0.05; **p < 0.001

The mRNA expressions of *Chop* and *Bip* significantly increased in the LPS, LPS+NEB, and NEB groups compared to the control group. However, in both the LPS+NEB and NEB groups, there was a significant decrease in *Chop* and *Bip* mRNA levels compared to the LPS group. Furthermore, in the NEB group, *Chop* mRNA levels significantly decreased, while *Bip* mRNA levels significantly increased compared to the LPS+NEB group (Fig. 1).

### Histopathological analysis

#### NEB ameliorates the pathological findings of liver tissues damaged by LPS

**Fig. 2:**
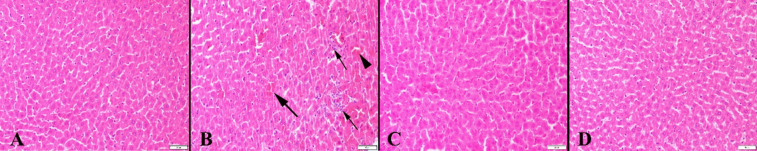
Histopathological appearance of livers between the groups. **A** Normal liver histology in the control group. **B** Marked hyperemia (thick arrow), a small hemorrhage (arrowhead), and inflammatory cell infiltrations in the LPS group. **C** Decreased pathological findings in the LPS+NEB group. **D** Normal tissue architecture in the NEB group, HE, scale bars = 50 µm

Normal liver histology was observed in the control and NEB groups during histological evaluation. Conversely, the LPS group displayed increased hyperemia, slight hemorrhages, hepatocyte degeneration, and inflammatory cell infiltrations, predominantly comprising neutrophil leukocytes. However, a reduction in these pathological findings was evident in the LPS+NEB group (Fig. 2).

#### NEB improves the immunohistochemical findings observed in liver tissue damaged by LPS

**Fig. 3:**
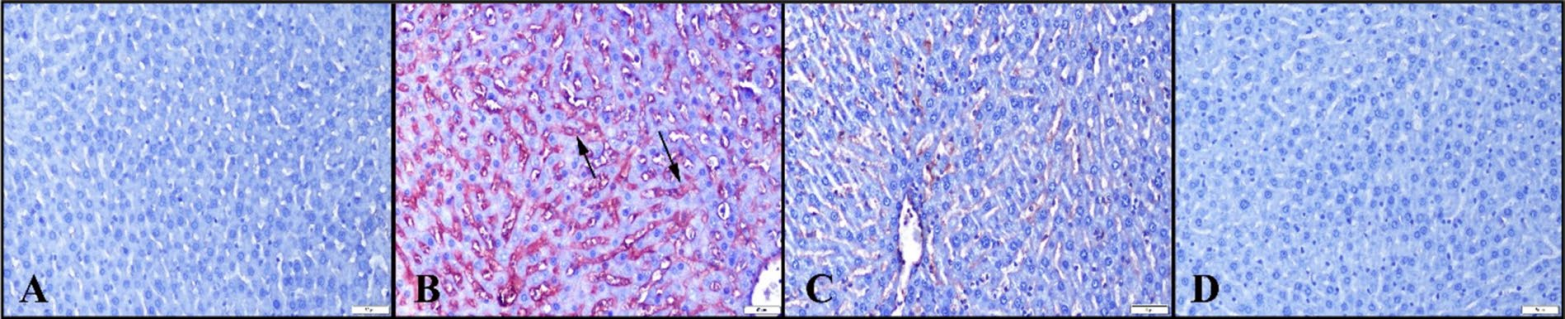
Cas-3 immunohistochemistry results between the groups. **A** Negative expression in the control group. **B** Marked increase in expression (arrows) in the LPS group. **C** Decreased expression in the LPS+NEB group. **D** No expression in the NEB group, Streptavidin-biotin peroxidase method, scale bars = 50 µm

**Fig. 4:**
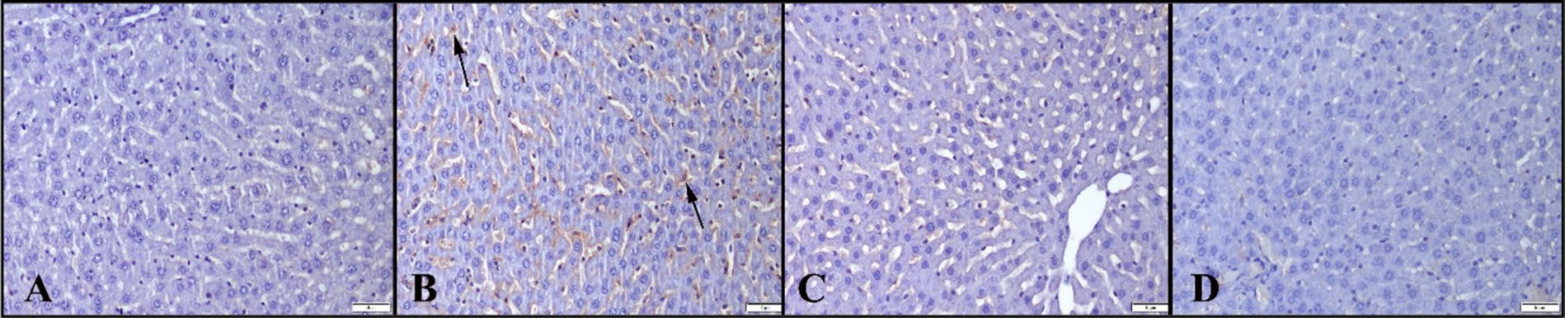
IL-1β immunoreactions between the groups. **A** No expression in the control group. **B** Marked expressions (arrows) in the LPS group. **C** Decreased expressions in the LPS+NEB group. **D** Negative expression in the NEB group; streptavidin-biotin-peroxidase method; scale bars = 50 µm

The immunohistochemistry analysis showed very slight or no expression of cas-3 or IL-1β in the control and NEB groups. In contrast, a significant increase was observed in the LPS group, notably expressed Kupffer cells, and hepatocytes (Figs. 3 and 4). Statistical analysis results of histopathological and immunohistochemical scores between the groups are shown in Table 4.

**Table 4:** Statistical analysis results of histopathological and immunohistochemical scores between the groups

Groups	Histopathology scores		Cas-3 IHC scores		IL-1β IHC scores	
	Mean ± SD	*p* value	Mean ± SD	*p* value	Mean ± SD	*p* value
Control	0.12 ± 0.12		0.12 ± 0.12		0.12 ± 0.12	
LPS	1.37 ± 0.50	0.001***	1.37 ± 0.51	0.001**	1.00 ± 0.75	0.01**
LPS+NEB	0.37 ± 0.18	0.001^###^	0.37 ± 0.18	0.001^###^	0.25 ± 0.16	0.01^###^
NEB	0.00 ± 0.00		0.12 ± 0.12		0.12 ± 0.12	

Values are presented as means ± SD. The relationships between groups and results of histopathological and immunohistochemical scores are assessed by one-way ANOVA test (post hoc LSD test)

*Cas-3* caspase-3, *IL-1β* interleukin 1β, *IHC* immunohistochemistry, *LPS* lipopolysaccharide, *NEB* nebivolol

^*^Compared to control group. *p ≤ 0.05, **p ≤ 0.01, ***p ≤ 0.001

^#^Compared to the LPS group, ^#^*p* ≤ 0.05, ^##^*p* ≤ 0.01, ^###^*p* ≤ 0.001

## Discussion

This experimental model of sepsis demonstrates that LPS administration increases the expression of *Chop*, *Bip/GRP78*, *IL-1β*, and caspase-3 in the rat liver. Notably, NEB treatment ameliorated LPS-induced liver injury and reversed the increase in AST, ALT, oxidative stress index, and TOS levels.

In systemic inflammatory diseases with multi-organ involvement, such as sepsis, progressive damage occurs in various tissues, including vital elimination organs such as the liver and kidneys. These damages give rise to many challenges in patient management, including metabolic dysfunction and excretory issues. In addition to oxidative stress, inflammation, and apoptosis, the autophagy process and ER stress mechanism, which are crucial for maintaining cellular homeostasis, contribute significantly to tissue damage. Numerous studies have revealed that these damage mechanisms can trigger one other (Liu et al. 2011; Zhang 2010). For example, ROS-induced oxidative stress can activate various intracellular pathways, leading to inflammation, and inflammatory cytokines, such as nuclear factor kappa B (NF-KB), can increase cell nucleus–mediated cas-3 synthesis, driving apoptosis. Additionally, excessive ER stress has been demonstrated to promote cas-3-mediated apoptosis through calpains (Morishima et al. 2002; Rathnasamy et al. 2014).

The parallel increase in oxidant substances and liver enzyme levels observed in this study supports these relationships. The liver, functioning as the body’s detoxification center, is exposed to high levels of oxidant substances. Under oxidative stress conditions, there is an increased consumption of antioxidant enzymes, which are essential defense mechanisms against the increased oxidative effects. Importantly, this hypothesis is further supported by the decrease in TAS levels in the injury group. A study assessing NEB treatment in rats with ischemia-reperfusion injury also found lower TAS levels in the injury group compared to the control and NEB treatment groups (Altunkaynak-Camca and Yazihan 2021). The increased TAS levels in the treatment group suggest that NEB decreases damage by increasing antioxidant enzyme production.

In the clinical evaluations of liver tissue damage, parameters like AST and ALT are commonly assessed, reflecting oxidative stress as a primary pathophysiological mechanism. Consistent with the findings of previous studies, rats in the LPS group exhibited a significant increase in serum AST and ALT levels, yet NEB treatment notably decreased these markers (Rofaeil et al. 2017; Ulger et al. 2015).

Inflammation, a crucial contributor to tissue damage, can be incited by heightened ROS levels or other cellular pathways (Soliman et al. 2019). This study investigated the impact of inhibiting ER stress, a distinct pathway, on inflammatory and apoptotic responses in the tissues. The reduction in IL-1β, an acute-phase reactant commonly used to monitor this response, following NEB administration in the injury group, signifies the drug’s anti-inflammatory effect. This observation aligns with decreased histopathologic signs like hyperemia, hemorrhage, and inflammatory cell infiltration in the tissues. Moreover, in a study examining the protective role of NEB in cadmium-induced hepatotoxicity through various pathways, the NEB treatment was observed to mitigate hepatocellular damage (Refaie et al. 2018).

Basal ER stress is crucial for maintaining cellular homeostasis. However, exceeding the ER’s capacity or disruptions in the protein quality control mechanism can impact other cellular stress processes. Elevated levels of *Chop*, a pro-apoptotic transcription factor, and *Bip* protein, a renowned ER chaperone, are directly linked to ER stress (Zhao and Ackerman 2006). Prolonged elevation in these protein levels can trigger apoptosis via an increase in *Cas-3* levels, a pro-apoptotic protein, mediated by calpain, an essential Ca^2+^-dependent protein (Liu et al. 2016). The present study demonstrated significant increases in the levels of *Chop* and *Bip* mRNA and strong immunohistochemical staining of cas-3 in the LPS-induced damage group, indicating the development of ER stress–mediated apoptosis in response to LPS-induced tissue damage. Consistent with the literature, analyses also revealed increased *Chop* mRNA and protein levels in inflammatory groups experiencing LPS-induced tissue damage (Esposito et al. 2013; Nakayama et al. 2010). Importantly, NEB treatment significantly mitigated apoptotic findings in the LPS groups, suggesting that NEB reverses LPS-induced ER stress. Moreover, the impact of the *Chop* gene may activate the internal mitochondrial pathway, leading to apoptosis. NEB’s inhibition of this pathway might also prevent mitochondrial stress. Other beta-blockers like carvedilol, propranolol, and atenolol have demonstrated ER stress alleviation in human coronary artery endothelial cells and hepatocyte-derived HepG2 cells, reducing endothelial cell ER and oxidative stress, potentially contributing to their hepatoprotective effects (Haas et al. 2016; Yang et al. 2018).

## Conclusion

This study successfully established an LPS-induced sepsis model in rats and demonstrated the effective mitigation of LPS-induced liver injury by NEB, a long-acting, third-generation beta-blocker targeting ER stress, oxidative stress, and apoptotic processes. Our findings significantly contribute to the existing literature, given the limited research on the relationship between NEB and ER stress. Considering, the implications of oxidative stress and ER in various disorders’ pathogenesis, this study suggests the promising therapeutic potential of NEB. However, future investigations exploring NEB’s potential protective impact against mitochondrial stress under similar pathological conditions would be beneficial.

### Supplementary Information

Below is the link to the electronic supplementary material.

Supplementary file1 (DOCX 114 KB)

## Data Availability

The data that support the findings of this study are available from the corresponding author, [OU], upon reasonable request.

## Data availability

Data supporting the findings of this study are available to any reader upon reasonable request.
