# Supplementary material for: Nebivolol protects the liver against lipopolysaccharide-induced oxidative stress, inflammation, and endoplasmic reticulum–related apoptosis through Chop and Bip/GRP78 signaling
Source: Naunyn Schmiedebergs Arch Pharmacol. 2024 Feb 14;397(8):5899–907. doi: 10.1007/s00210-024-02990-3 (PMC11329546; doi:10.1007/s00210-024-02990-3)
Supplement: Supplementary file 1 — Supplementary file1 (DOCX 114 KB) [file 210_2024_2990_MOESM1_ESM.docx]

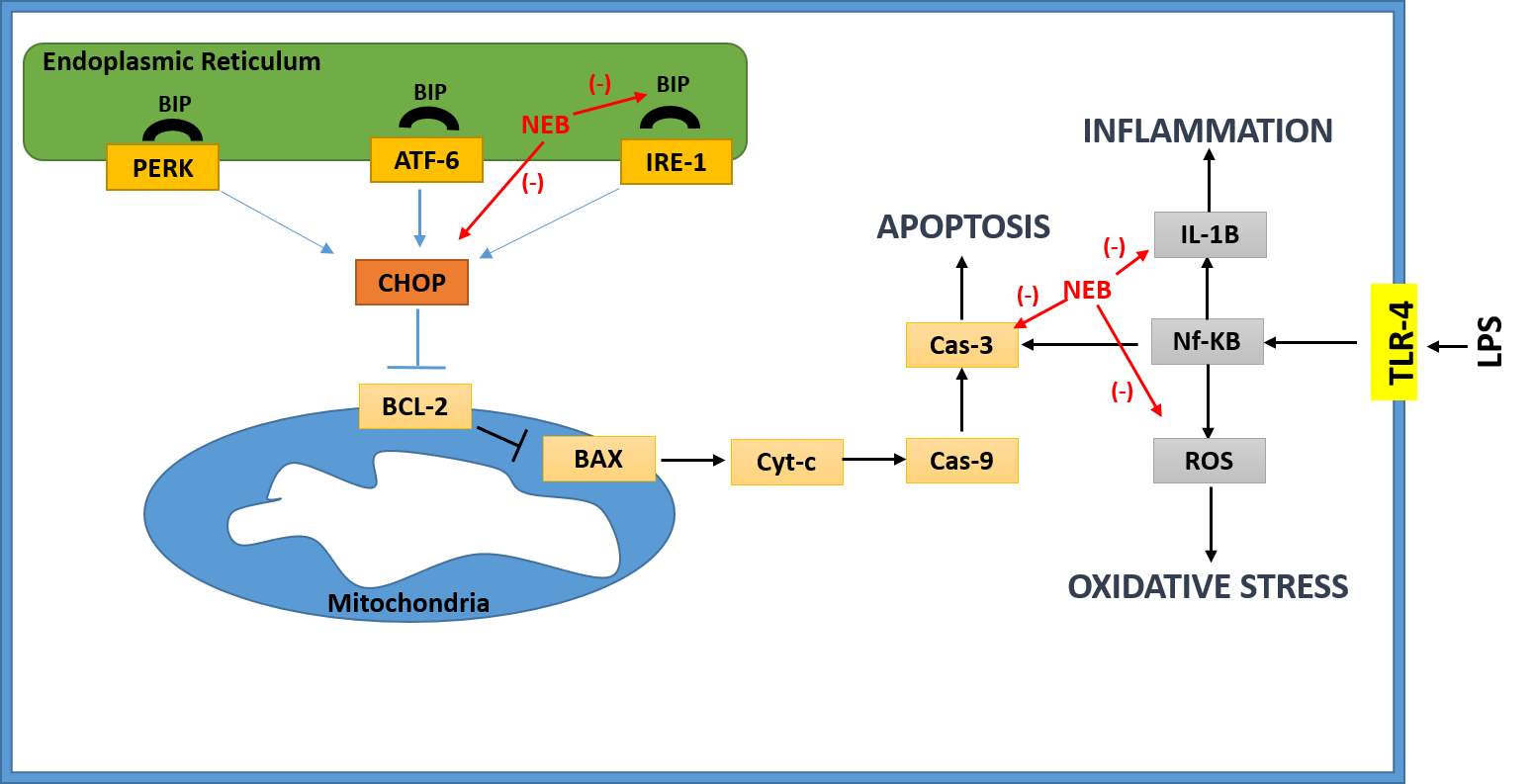


**Supplementary material:**The action mechanism of Nebivolol on LPS induces liver toxicity

**Cas-3:** Caspase-3, **Cas-9:** Caspase-9, **IL-1B:** interleukin-1 beta, **NF-κB:** nuclear factor kappa B, **CHOP:** CCAAT-enhancer-binding protein homologous protein, **ATF6:** Activating transcription factor 6, **PERK:** protein kinase RNA-like endoplasmic reticulum kinase, **IRE-1:** Inositol-Requiring Enzyme 1, **ROS:** reactive oxygen species, **BAX:** Bcl-2-associated X protein, **BCL-2:** B-cell lymphoma protein 2, **Cyt-c:** cytochrome c
